# Synthetic Glycopolypeptide Micelle for Targeted Drug Delivery to Hepatic Carcinoma

**DOI:** 10.3390/polym10060611

**Published:** 2018-06-04

**Authors:** Pengqiang Li, Jiandong Han, Di Li, Jinjin Chen, Wei Wang, Weiguo Xu

**Affiliations:** 1 Department of Chemistry, Changchun University of Science and Technology, Changchun 130022, China; pqlicn@outlook.com (P.L.); jdhan@ciac.ac.cn (J.H.); 2 Key Laboratory of Polymer Ecomaterials, Changchun Institute of Applied Chemistry, Chinese Academy of Sciences, Changchun 130022, China; lidi@ciac.ac.cn (D.L.); jjchen@ciac.ac.cn (J.C.)

**Keywords:** glycopolypeptide, micelle, targeting chemotherapy, controlled drug release, hepatic carcinoma

## Abstract

The targeted delivery of chemotherapy drugs to tumor lesions is a major challenge for the treatment of tumors. Up until now, various polymeric nanoparticles have been explored to improve the targetability of these therapeutic drugs through passive or active targeting processes. In the design and construction of polymer nanoparticles, glycopolypeptide has shown great potential owing to its excellent targeting ability and biocompatibility. In order to enhance the antitumor effect of doxorubicin (DOX), a glycopolypeptide-based micelle (GPM) modified by α-lactose (Lac) was synthesized for targeted treatment of hepatoma. The DOX-loaded GPM (i.e., GPM/DOX) could significantly target human hepatoma (HepG2) cells and further inhibit their proliferation *in vitro*. Additionally, GPM/DOX exhibited a much higher drug accumulation in tumor tissue and a stronger antitumor effect *in vivo* than free DOX. The above results revealed that this drug delivery system provides a promising platform for the targeting therapy of hepatic cancer.

## 1. Introduction

Chemotherapy is widely applied in cancer treatment and exhibits particular superiority compared with surgery and radiotherapy. Though drugs with different mechanisms have been developed for various malignancies, patients still suffer severe side effects due to the undesired distribution of free drugs in normal organs. Doxorubicin (DOX), for example, is a broad-spectrum chemotherapy drug applied in many types of malignancies, e.g., hepatic cancer, breast cancer, ovarian cancer, lung cancer, and soft tissue sarcoma [1]. However, the antitumor application of DOX is largely hindered by its significant defects, such as poor target character, low solubility, short blood circulation, heart damage, and so on [2]. By contrast, targeted chemotherapeutic systems that can not only increase the drug accumulation at tumor sites but also decrease the drug distribution in normal organs are needed urgently [3,4].

To this end, nanomedicine has come to light as a potential platform for targeting the delivery of chemotherapeutics through passive and/or active targeting [5]. First, the nano-sized vehicles can accumulate at tumor sites through enhanced permeability and retention (EPR) effects; second, after being modified with specific ligands, these nanocarriers can recognize the tumor cells through specific ligand–receptor interaction [6,7]. This can provides an opportunity to overcome the intrinsic limits of conventional cancer therapies [8]. In recent years, glycopolypeptide has attracted a great deal of attention in medical materials because of its unique molecular composition and similar structure to natural glycoproteins [9,10]. Usually, oligosaccharides served as signal molecules at the end of glycoproteins [11]. At the same time, the presence of oligosaccharides prevents the proteases from directly contacting the polypeptide to reduce the degradation of glycopolypeptide and improve the stability of the materials [12]. More importantly, some monosaccharides or polysaccharides, such as α-lactose (Lac), sialic acid (SA), hyaluronic acid (HA), show the ability to target tumor cells [13]. Thus, glycopolypeptide might be an ideal candidate for targeted chemotherapy [14].

## 2. Materials and Methods

The materials and methods were listed in Supplementary Material.

## 3. Results and Discussion

### 3.1. Preparation and Characterization of Loading GPM Micelles

**Scheme 1:**
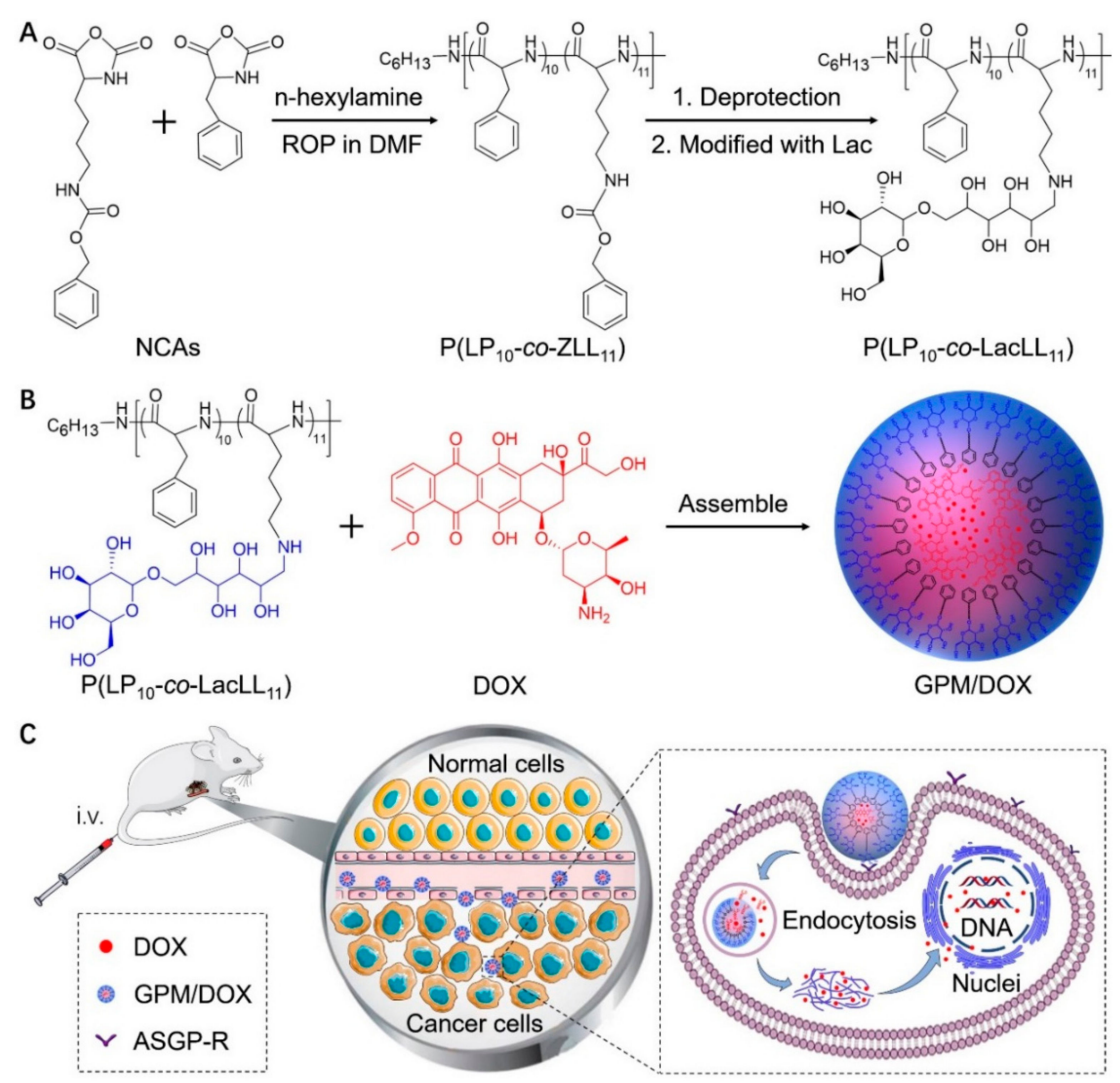
DOX-loaded glycopolypeptide-based micelle (i.e., GPM/DOX) synthesis and self-assembly diagram (**A**,**B**), and intravenous injection, *in vivo* circulation, tumor target accumulation, and the release of DOX in cells (**C**).

In this study, a glycopolypeptide-based micelle (GPM), modified with Lac, was prepared for targeted chemotherapy. First, poly(l-phenylalanine-co-γ-benzyl-l-lysine) (P(LP10-co-ZLL_11_)) was synthesized by ring-opening polymerization (ROP) of LP *N*-carboxyanhydride (LP NCA) and ZLL *N*-carboxyanhydride (ZLL NCA) using n-hexylamine as the initiator. Then, the polypeptide was deprotected to obtain the cationic copolymer, poly(LP-*co*-l-lysine) (P(LP_10_-*co*-LL_11_)). Second, the Lac was conjugated to the amino group for specific recognition of hepatic cells by selectively binding to asialoglycoprotein receptor (ASGP-R) (Scheme 1A). Galactose/*N*-acetylglucosamine groups could identify the ASGP-R overexpressed on the surface of hepatoma carcinoma cell and play an important role in receptor-mediated endocytosis [15,16,17].

Finally, the glycopolypeptide could self-assemble into nano-sized micelles via hydrophobic interaction to act as a carrier. Free DOX was chosen as a model drug and then loaded into the core of GPM through strong π-π interaction with the phenylalanine block (Scheme 1B) [18,19,20]. The DOX- loaded GPM (i.e., GPM/DOX) showed superior properties compared with free DOX: (1) Owing to the suitable size, GPM/DOX could accumulate at the tumor sites through EPR effect; (2) through the π-π stacking effect, the drug loading content (DLC) of GPM was obviously improved; (3) the polymer micelle could target hepatic cells actively through selective recognition between Lac and ASGP-R (Scheme 1C).

**Figure 1:**
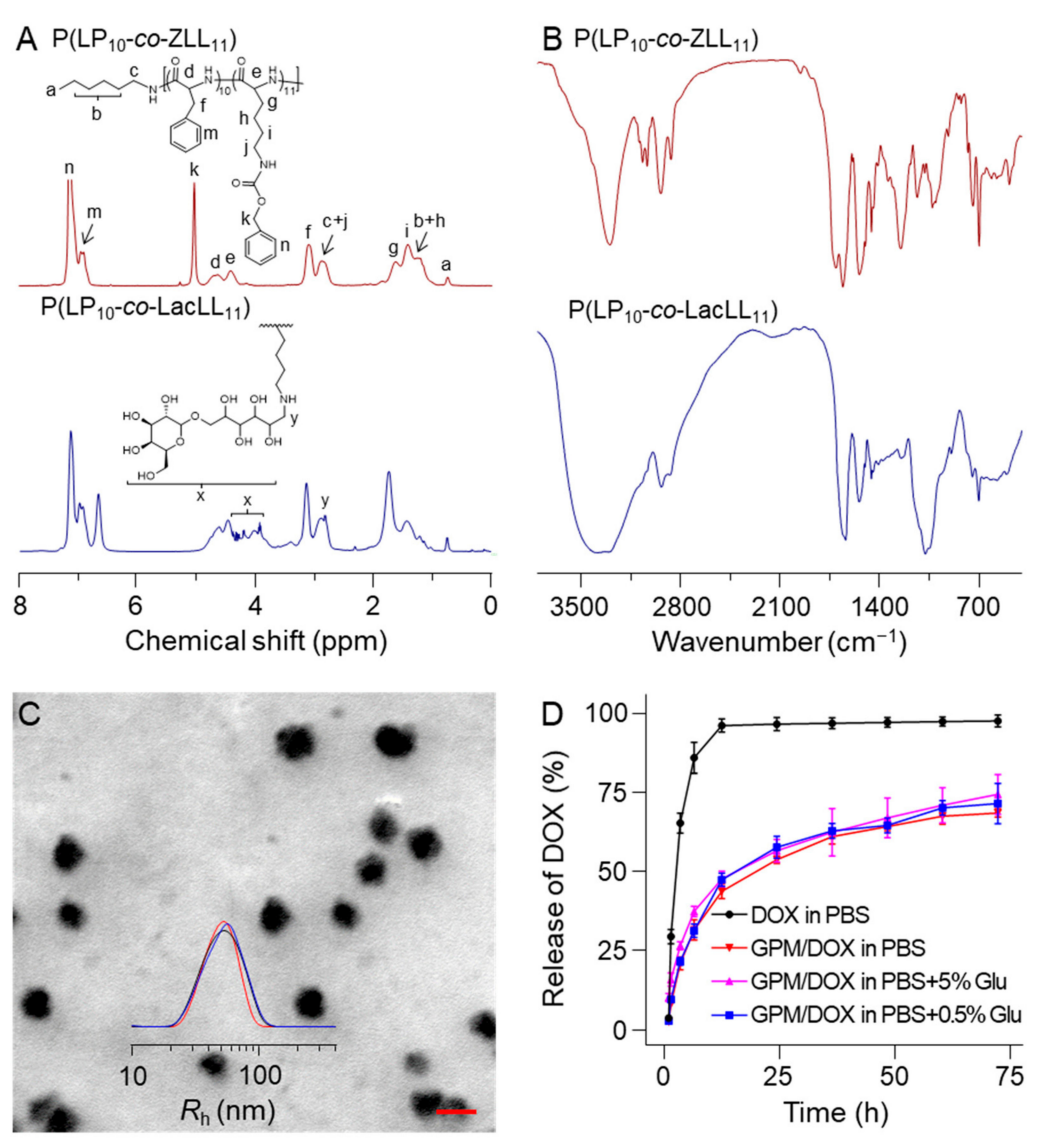
(**A**) ^1^H NMR (in TFA-*d*); (**B**) FT-IR spectra of P(LP_10_-*co*-ZLL_11_), P(LP_10_-*co*-LL_11_), and P(LP_10_-*co*-LacLL_11_); (**C**) SEM microimages and *R*_h_ of GPM; and (**D**) release of free DOX and GPM/DOX in PBS containing different concentrations of Glu. Here, each set of data is represented as mean ± standard deviation (SD) (*n* = 3); scale bar = 100 nm.

The chemical structures of these polymers were confirmed with a 500 MHz Avance III HD nuclear magnetic resonance (NMR, Rheinstetten, Germany) spectrometer and a fourier transform infrared spectrometer (FT-IR; Bio-Rad, FTS-600; Cambridge, MA, USA). As shown in ^1^H NMR spectra (Figure 1A), for P(LP_10_-*co*-ZLL_11_), the peak at 0.81 ppm belonged to the protons in methyl of the n-hexylamine initiator. The strong peak at 5.10 ppm was assigned to the protons in methylene of benzyloxycarbonyl. The peaks at 4.49 and 4.70 ppm were attributed to the protons of methine on the polypeptide backbone. The signals from 6.98 to 7.28 ppm were typical symbols of benzene. After deprotection, the spectrometer was shown in Figure S1. All these above results indicated that the P(LP_10_-*co*-ZLL_11_) was successfully synthesized. Furthermore, the emergence of characteristic peaks of Lac from 3.51 to 4.39 ppm in TFA-*d* and the peak at 2.98 ppm indicated the successful synthesis of glycopolypeptide. In FT-IR spectra (Figure 1B), 1656 (*V*_C=O_) and 1703 cm^−^^1^ (*V*_C(O)–NH_) proved the successful generation of the polypeptide block. After deprotection, the absorption peak at 3300 cm^−1^ appeared owing to the exposure of amino group in P(LP_10_-*co*-LL_11_) (Figure S2). Finally, the signals at 1076 cm^−1^ were assigned to Lac, demonstrating the successful preparation of glycopolypeptide P(LP_10_-*co*-LacLL_11_).

The GPM/DOX were prepared by mixing the as-prepared glycopolypeptide with DOX in Milli-Q water and then stirring in the dark at room temperature for 24 h. Afterwards, the free DOX was removed by the dialysis methods. The final solution was filtrated followed by lyophilization. The drug loading content (DLC) and the drug loading efficiency (DLE) of GPM/DOX were determined to be 9.6 wt % and 51.4 wt % by an ultraviolet-visible (UV-Vis) spectrophotometer (UV-1800; Shimadzu, Kyoto, Japan) at the wavelength of 480 nm. The zeta potential of the GPM/DOX was measured to be 6.6 ± 1.4 mV in water using a Zeta Potential Analyzer (ZETA PALS; Brookhaven instruments corporation, Holtsville, NY, USA).

The glycopolypeptide could self-assemble into stable micelles in an aqueous solution, and the critical micellar concentration (CMC) of GPM was determined to be 33.4 μg mL^−1^ by fluorescence spectra using pyrene as a probe (Figure S3). The transmission electron microscopy (TEM; JEOL Ltd., Tokyo, Japan) images showed that the GPM/DOX exhibited a near-spherical morphology with an average diameter of about 53.0 nm Furthermore, the aqueous size of GPM/DOX was measured by dynamic light scattering (DLS) for three times, as shown in Figure 1C, the micelles had a uniform radius of 53.7 ± 9.2 nm (*n* = 3) in PBS solution (pH 7.4).

The *in vitro* release behaviors of DOX-load micelles were monitored in PBS with different concentrations of glucose (Glu). Specifically, the pure PBS is the preparation condition for a drug delivery system; 5% or 0.5% PBS-buffered Glu solution is commonly used in clinical research. As shown in Figure 1D, for free DOX, more than 97.0% DOX was detected in the initial 12 h. In contrast, for GPM/DOX, almost 22% of the drug was released within the first three hours and the cumulative release amount of DOX after 12 h was less than 45.0% in three different solutions. The sustained release behavior of GPM/DOX continued to do so more than 72 h. This characteristic could help to reduce the side effects caused by the fast distribution of free DOX *in vivo*. It was worth mentioning that the release behavior showed no difference between 5% and 0.5% PBS-buffered Glu solution, which indicated that the GPM/DOX had the potential of application in clinical therapy.

### 3.2. Cell Internalization and Cell Proliferation Inhibition

**Figure 2:**
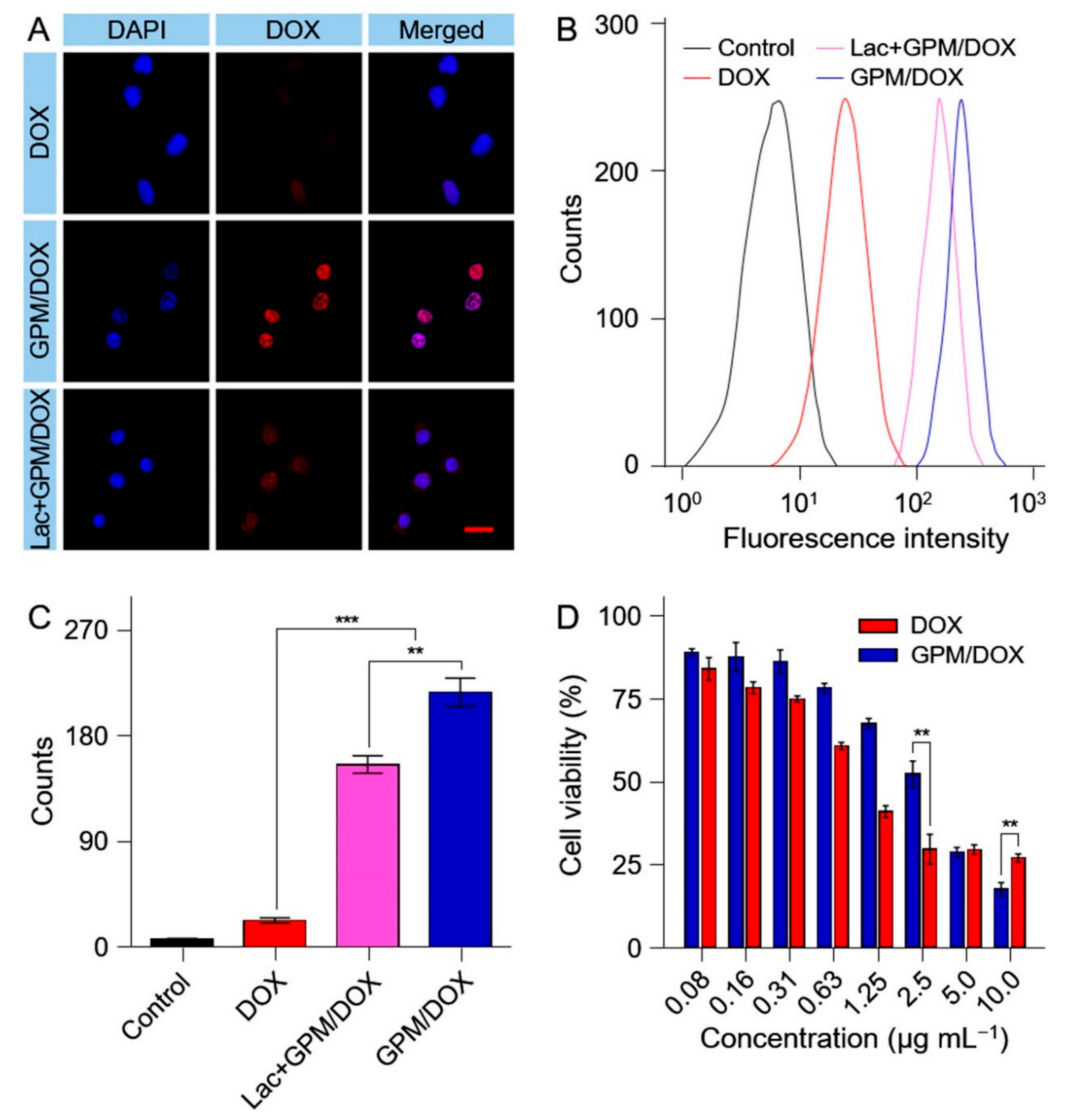
Cellular uptakes and intracellular DOX release of GPM/DOX and free DOX after incubation with HepG2 cells for 2 h detected by (**A**) Confocal laser scanning microscopy (CLSM) and (**B**) Flow cytometry method (FCW) analyses; (**C**) The quantization of FCM; (**D**) *In vitro* cytotoxicities of GPM/DOX and free DOX after incubation with HepG2 cells for 24 h; data were presented as a mean ± SD (*n* = 5; ** *p* < 0.01, *** *p* < 0.001); scale bar = 50 μm.

The cell uptake of GPM/DOX by HepG2 cells were evaluated through confocal laser scanning microscopy (CLSM, LSM 780; Carl Zeiss, Oberkochen, Germany) and the flow cytometry method (FCM). Specifically, HepG2 cells were incubated with free DOX, GPM/DOX, or GPM/DOX with Lac at an equivalent DOX concentration of 5.0 μg mL^−1^ for 2 h. As shown in Figure 2A, the fluorescence intensity of DOX in the GPM/DOX group was stronger than that in DOX group, which indicated GPM/DOX could target HepG2 cells due to the decoration with Lac. However, the cell uptake of GPM/DOX was markedly decreased when the ASGP-R on the HepG2 cell surfaces was occupied by free Lac at 2.0 mg mL^−1^. A similar result was observed by FCM analysis (Figure 2B,C). All results further confirmed that the targeting ability of GPM/DOX was given by the recognition of Lac group.

The proliferation inhibition effects of free DOX and GPM/DOX toward HepG2 cells were evaluated by 3-(4, 5-dimethyl-2-thiazolyl)-2, 5-diphenyl-2-H-tetrazolium bromide (MTT) assay *in vitro*. The cells were incubated with GPM/DOX or free DOX at various concentrations of DOX from 0.08 to 10 μg mL^−1^. After incubation for 24 h, GPM/DOX showed a stronger cytotoxicity than free DOX (Figure 2D). The half maximal inhibitory concentrations (IC_50_s) of free DOX and GPM/DOX were further calculated to be 1.2 and 2.0 μg mL^−1^, respectively. The ability to target tumor cells and endocytosis gave GPM/DOX more toxic to the tumor cells.

### 3.3. Biodistribution

**Figure 3:**
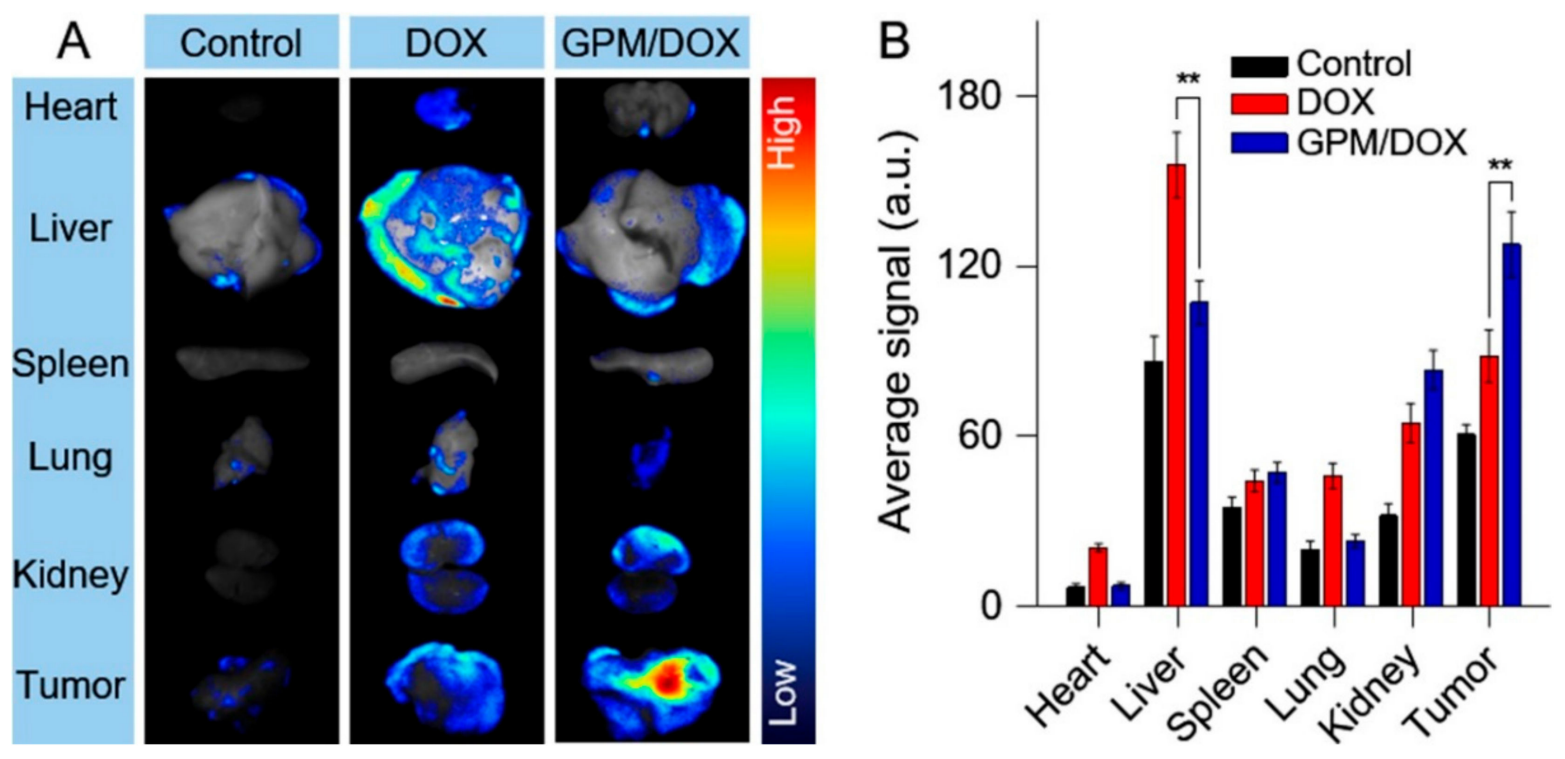
(**A**) Ex vivo DOX fluorescence images showing the semiquantitative tissue distribution of DOX; and (**B**) the average strength collected from the major organs (i.e., heart, liver, spleen, lung, and kidney) and tumors after i.v. injection of normal saline, GPM/DOX, or free DOX to H22-xenografted Kunming mice for 12 h; data were presented as a mean ± SD (*n* = 3; ** *p* < 0.05).

The evaluation of biodistribution is the key step to determine whether a new drug can be applied in clinic or not [5]. In this study, all animals were provided by Charles River laboratories (Beijing, P. R. China). At the same time, all animals received care in compliance with the guidelines outlined in the Guide for the Care and Use of Laboratory Animals, and all procedures were approved by the Animal Care and Use Committee of Jilin University. Kunming mice bearing H22 hepatocellular carcinoma were sacrificed 12 h after drug injection, and tumors and vital organs were isolated for fluorescence imaging (Figure 3A). The DOX fluorescence in the liver was higher in the mice of the free DOX group than those in the GPM/DOX group, which revealed that free DOX was metabolized more quickly by the liver. Likewise, more DOX distribution was observed in the hearts and kidneys of mice treated with free DOX as an indicator of stronger toxicity toward these two organs. However, less fluorescence intensity in the heart or kidney was found in the GPM/DOX group. It was worth noticing that the mice administered with the GPM/DOX micelle showed a much stronger fluorescence intensity of DOX at tumor sites than those in the free DOX group. In addition, the fluorescence intensity was semi-quantitatively analyzed by Maestro™ 2.4 software (Cambridge Research & Instrumentation, Inc., Hopkinton, MA, USA). As shown in Figure 3B, the DOX fluorescence intensity of tumors in the GPM/DOX group was 1.5 times that of free DOX group. Consistent with the previous findings, the fluorescence imaging test further confirmed the selectively intratumoral accumulation of GPM/DOX.

### 3.4. In Vivo Antitumor Efficacy

**Figure 4:**
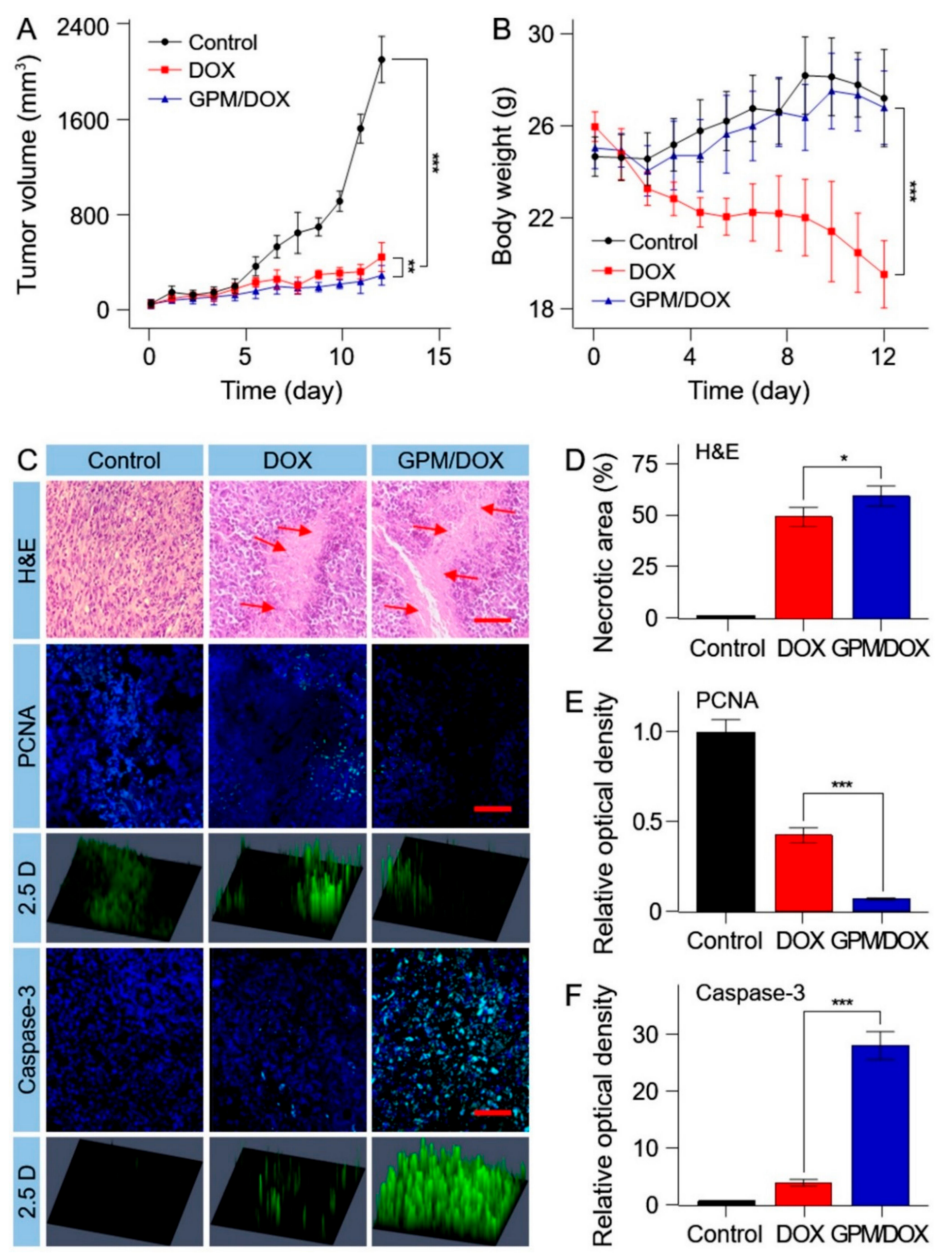
(**A**) Tumor volumes; (**B**) body weights of H22-xenografted mice after treatment with GPM/DOX or free DOX·HCl with normal saline as control; data were presented as mean ± SD (*n* = 8; ** *p* < 0.01, *** p < 0.001); (**C**) histopathological (i.e., H&E), immunohistochemical (i.e*.*, Caspase-3 and PCNA) after treatment with GPM/DOX, free DOX, or normal saline (as control). Scale bar = 100 μm ×, for H&E, Caspase-3 and PCNA; (**D**–**F**) quantitative analysis of H&E, PCNA, and Caspase-3; data were presented as mean ± SD (*n* = 8; * *p* < 0.05, *** *p* < 0.001).

Assessment of anti-tumor potential *in vivo* is an essential part of preclinical study of novel antitumor agents [21,22]. In this study, the *in vivo* antitumor effect of GPM/DOX was carried out in BALB/c mice bearing subcutaneous H22 hepatic tumors. When the tumor volume reached about 80 mm^3^, mice were treated with free DOX, GPM/DOX, or saline as control by tail vein injection every 4 days at a dose of 5 mg kg^−1^ per body weight. The tumor volumes were continuously observed and recorded for 12 days before the mice were sacrificed. As shown in Figure 4A, both free DOX and GPM/DOX could suppress the expansion of tumor volume to some extent. The tumor inhibition rates of DOX and GPM/DOX were 77.7% and 85.0%, respectively, which demonstrated that GPM/DOX achieved a better therapeutic effect than free DOX. The suitable particle size and targeting ability contributed to a remarkable antitumor efficacy of GPM/DOX. As shown in Figure 4B, the body weights of mice treated with free DOX decreased dramatically, whereas the mice of the GPM/DOX group maintained a similar body weight to the control group, proving that GPM/DOX could reduce the toxicity of chemotherapy significantly.

The antitumor effects of GPM/DOX were verified by histopathology and immunohistochemistry (IHC) analyses in mice tumor tissues. The tumor tissue sections were prepared with hematoxylineosin (H&E) staining. As shown in Figure 4C (H&E), the tumor cells of the control group grew actively, while the tumor cells began to become apoptotic in the free DOX and GPM/DOX groups. Furthermore, the tumor necrosis areas were the largest in all groups, and it was up to 63.6 ± 5.2% and about 1.2-fold larger than that of free DOX group (Figure 4D). The data of the tumor necrosis areas were consistent with tumor inhibition rates in general. A proliferating cell nuclear antigen (PCNA) stain was used to assess the tumor cell proliferation ability (Figure 4C). As shown in Figure 4E, the expressions of PCNA in free DOX was 5.8-fold higher than that in GPM/DOX group. These signals would be more clearly seen in the 2.5D phase. Caspase-3 is commonly activated by numerous death signals. As shown in Figure 4C,F, the expression of Caspased-3 showed a contrary tendency with that of the PCNA sections, and the same experiment result was proved once again. Based on the findings above, it was demonstrated that the synthetic GPM/DOX had a better effect on inhibiting tumor proliferation and promoting tumor apoptosis than free DOX.

### 3.5. In Vivo Antitumor Efficacy

The *in vivo* safety of GPM/DOX were further studied by histopathology. The pathological sections of main organs were stained with H&E. As shown in Figure S4, the free DOX and GPM/DOX groups exhibited pathological changes in H&E to some extent. The main changes are as follows: (i) destruction of myocardial structure and inflammation; (ii) the renal capsule cavity shrank or disappeared. The damage levels to the hearts or kidneys of mice in the free DOX group were more serious than those in the GPM/DOX group, which further confirmed that GPM/DOX had higher safety than free DOX.

## 4. Conclusions

In conclusion, a glycopolypeptide-based micelle was prepared for targeted chemotherapy of hepatic malignancy. The GPM/DOX could enhance drug accumulation in tumors and reduce the distribution in nontargeted tissues. In particular, the drug concentration in heart in GPM/DOX groups were only one third of that in the free DOX group, significantly reducing cardiotoxicity of DOX, which certainly showed an excellent antitumor effect and a high safety *in vivo*. The simple process and good performance of GPM/DOX provide a possibility for clinical application.

## Supplementary Materials

The following are available online at http://www.mdpi.com/2073-4360/10/6/611/s1: Materials, full experimental details including synthesis, physicochemical characterization, *in vitro* cytotoxicity, cell apoptosis analyses, cell immunohistochemical staining, antitumor assays in vivo, *in vivo* antitumor efficacy, and systemic toxicity. All experimental details are available in the online version of the paper.
