# Supplementary material for: Synthetic Glycopolypeptide Micelle for Targeted Drug Delivery to Hepatic Carcinoma
Source: Polymers (Basel). 2018 Jun 4;10(6):611. doi: 10.3390/polym10060611 (PMC6403909; doi:10.3390/polym10060611)
Supplement: Supplementary file 1 [file polymers-10-00611-s001.pdf]

Supplementary information

Synthetic glycopolypeptide micelle for targeted drug delivery to hepatic carcinoma

Pengqiang Li[1,2†], Jiandong Han[1,2†], Di Li[2], Jinjin Chen[2], Wei Wang[1*], Weiguo Xu[2*]

1. Department of Chemistry, Changchun University of Science and Technology, Changchun 130022, China
2. Key Laboratory of Polymer Ecomaterials, Changchun Institute of Applied Chemistry, Chinese Academy of Sciences, Changchun 130022, China
E-mail: weiwanglg@163.com (W.W.); *wgxu@ciac.ac.cn* (W.X.)

Contents:

1. Materials and Methods
2. Supplementary figures
Figure S1 ^{1}H NMR spectra of P(LP$_{10}$-*co*-LL$_{11}$)
Figure S2 FT-IR spectra of P(LP$_{10}$-*co*-LL$_{11}$)
Figure S3 CMC of GPM
Figure S4 *Ex vivo* histological analyses of main organ sections from H22-xenografted mice

1.1 Materials

α-lactose monohydrate (Lac; Number-average Molecular Weight (M_n) = 360.31 Da), sodium cyanoborohydride ($NaBH_3CN$; M_n = 62.84); hydrobromic acid/acetic acid solution (HBr/HAc, 33.0 wt.%) and n-hexylamine (M_n = 101.19 Da) were purchased from sigma-aldrich. Doxorubicin hydrochloride (DOX·HCl; Mn = 579.98) was obtained from Beijing HuaFeng United Technology Co., Ltd. (Beijing, P. R. China). L-Phenylalanine (LP; M_n=165.19 Da) and N_6-Cbz-L-Lysine (ZLL; M_n = 280.32 Da) were purchased from GL Biochem (Shanghai) Ltd. (Shanghai, P. R. China). Other reagents and solvents were purchased from Sinopharm Chemical Reagent Co. Ltd. and used as received. Cell culture products including Dulbecco's modified Eagle's medium (DMEM) and fetal bovine serum (FBS) were provided by Gibco (Gland Island, NY, USA). Caspase-3 and proliferating cell nuclear antigen (PCNA) were purchased from Abcam Company (Cambridge, UK). Penicillin and streptomycin were obtained from Huabei Pharmaceutical Co., Ltd. (Shijiazhuang, P. R. China). 3-(4,5-Dimethyl-thiazol-2-yl)-2,5-diphenyl tetrazolium bromide (MTT) and 4’,6-diamidino-2-phenylindole dihydrochloride (DAPI) were purchased Sigma-Aldrich (Shanghai, P. R. China). Human hepatoma HepG2 cells were obtained from North Carolina Chuang Lian Biotechnology Research Institute (Beijing, P. R. China). BALB/c mice were obtained from Beijing Vital River Laboratory Animal Technology Co., Ltd. and used at 5 weeks of age.

1.2 Methods

1.2.1. Synthesis of poly(L-phenylalanine-*co*-γ-benzyl-L-lysine) (P(LP_{10}-*co*-ZLL_{11}))

As shown in Scheme 1A in the text, poly(L-phenylalanine-*co*-γ-benzyl-L-lysine) (P(LP_{10}-*co*-ZLL_{11})) was obtained by the ring-opening polymerization (ROP) of LP NCA and ZLL NCA using n-hexylamine as an initiator. Specifically, dried LP NCA (2.3 g, 12 mmol) and ZLL NCA (3.7 g, 12.0 mmol) were dissolved in 50.0 mL of dried DMF in a dried flask, then, added n-hexylamine (131.3 μL, 1.0 mmol) under nitrogen protection. The mixture was stirred for three days at room temperature. Then, the solution was precipitated into 400.0 mL of diethyl ether. The obtained solid was dried under vacuum at room temperature ((P(LP_{10}-*co*-ZLL_{11}); Yield: 89.5%).

1.2.2 Synthesis of poly(L-phenylalanine-*co*-Lac-L-lysine) (P(LP_{10}-*co*-$LacLL_{11}$))

Firstly, removed the benzyloxycarbonyl group of P(LP_{10}-*co*-ZLL_{11}). Briefly, 2.5 g of P(LP_{10}-*co*-ZLL_{11}) was dissolved in 25.0 mL of TFA. Then, 7.5 mL of 33.3 wt.% HBr/HAc solution was added. After stirred for 1.5 h at room temperature, the solution was precipitated into 250.0 mL of diethyl ether. The obtained product was dissolved in water, dialyzed (molecular weight cut-off (MWCO) =

2000 Da) against water for three days, and then lyophilized to obtain solid (($P(LP_{10}$-*co*-$LL_{11})$; Yield: 85.2 %).

Secondly, modified $P(LP_{10}$-*co*-$LL_{11})$ with Lac. In detail, the $P(LP_{10}$-*co*-$LL_{11})$ (1.0 g; 0.3 mmol) was dissolved in morpholine ethanesulfonic acid solution (MES) and adjusted the pH to 5.5. Then, added Lac (26.7 g; 74.2 mmol) and $NaBH_3CN$ (7.0 g; 111.1 mmol) kept the pH in the range of 5.0−5.5 and the solution was stirred for three days at room temperature. Then, the solution was dialyzed (MWCO = 3500 Da) against water for three days and lyophilized to obtain white powder ($P(LP_{10}$-*co*-$LacLL_{11})$; Yield: 79.8%).

1.2.2. Proton nuclear magnetic resonance (^{1}H NMR) and Fourier-transform infrared spectroscopy (FT-IR) detections

^{1}H NMR spectra were recorded on a Bruker AV 500 NMR spectrometer in deuterated trifluoroacetic acid. FT-IR spectra were recorded on a Bio-Rad Win-IR instrument using potassium bromide method.

1.2.3. Dynamic laser scattering (DLS) and transmission electron microscopy (TEM) measurements

DLS measurements were performed with a vertically polarized He-Ne laser (DAWN EOS, Wyatt Technology). TEM assays were performed on a JEOL JEM-1011 transmission electron microscope with an accelerating voltage of 100 kV.

1.2.4. Critical micelle concentration (CMC) of glycopolypeptide micelle (GPM)

GPM was characterized by fluorescence spectroscopy using pyrene as a probe of the local hydrophobicity, which enables measurement of the CMC of GPM. The ratio of the fluorescence emission peaks ($I_{335.5}$ / $I_{338.5}$) were plotted over a range of GPM concentrations. The abscissa of the curve's inflection point was considered the critical micelle concentration.

1.2.5 The drug loading content (DLC) and drug loading efficiency (DLE) was calculated by Equation (1) and (2).

$$\text{DLC (wt.\%)} = \frac{\text{Weight of Drug in Micelle}}{\text{Weight of Drug-Loaded Micelle}} \times 100\% \quad (1)$$

$$\text{DLE (wt.\%)} = \frac{\text{Weight of Drug in Micelle}}{\text{Total Weight of Feeding Drug}} \times 100\% \quad (2)$$

The mass of DOX in GPM/DOX was determined using ultraviolet-visible (UV-vis) spectrophotometry with λ_{ab} = 480 nm (UV-1800, Shimadzu, Kyoto, Japan).

1.2.5. *In vitro* DOX release

The DOX release from GPM/DOX was tested in phosphate-buffered saline (PBS) at different

concentrations of different glucose concentrations (*i.e,* 0.5% and 5% (W/V), mimicking the preprandial blood glucose level in healthy human body and isotonic glucose injection in clinic, respectively at pH 7.4, 37 °C. GPM/DOX was weighted and dissolved in release medium with a concentration of 0.2 mg mL^{-1}. In detail, 10.0 mL of diluted solution was transferred into a dialysis bag (molecular weight cut off (MWCO) = 3500 Da). The release experiment was initiated by placing the end-sealed dialysis bag into 100.0 mL of release medium at 37 °C with 75 rpm constant shaking. At preselected time, 2.0 mL of the external buffer was collected and replenished with a fresh one. The amount of released DOX was determined by UV-vis spectrophotometer using a standard curve method.

1.2.6. Cell culture

HepG2 cells were cultured in complete DMEM supplemented with 10% (*V*/*V*) FBS, penicillin (50.0 IU mL^{-1}), and streptomycin (50.0 IU mL^{-1}) at 37 °C in a 5% (*V*/*V*) carbon dioxide (CO_2) atmosphere.

1.2.7. Cellular uptakes

The cells were seeded in 6-well plates with a density of 2.0×10^5 cells per well in 2.0 mL of complete DMEM and conventionally cultured for 12 h. Subsequently, the cells were cultured in conventional culture condition for 12 h. The original medium was replaced with GPM/DOX or DOX solution in complete DMEM at a final DOX concentration of 5.0 μg mL^{-1}. The cells were further conventionally incubated for 2 h, and then washed with PBS 3 times. The harvested cells were suspended in PBS and centrifuged at 1500 rpm for 6 min at 4 °C. The supernatants were discarded, and the cells were washed again with PBS to remove background fluorescence from the medium, repeat washing twice. The cells were resuspended in 500.0 μL of PBS. Data was analyzed by FCM (Beckman, California, USA).

1.2.8. Cytotoxicity assays

The cytotoxicities of GPM/DOX and DOX were evaluated by a MTT assay. HepG2 cells with a density of 8,000 cells per well were seeded in 96-well plates in 200.0 μL of complete DMEM and incubated for 12 h. The culture medium was replaced with 180.0 μL of fresh medium containing GPM/DOX and DOX with DOX (10.0 μg mL^{-1}). After 48 h incubation, the cells were subjected to MTT assay and then 20.0 μL of a stock solution containing 0.05 mg mL^{-1} of MTT in PBS was added and incubated for further 4 h. Then, the medium was replaced with 150.0 μL of DMSO. The

absorbance of above solution was measured on a Bio-Rad 680 microplate reader at 490 nm. The cell viability was calculated based on Equation (2).

$$\text{Cell viability (\%)} = \frac{A_{\text{sample}}}{A_{\text{control}}} \times 100 \qquad (3)$$

1.2.9. *In vivo* antitumor assays

The tumor-bearing mice were prepared through the subcutaneous injection in the armpits of right anterior limbs with 0.1 mL of cell suspension containing 2.0 × 10^6 mouse hepatocarcinoma H22 cells in PBS. The antitumor tests were performed when the tumor volumes grew up to around 80 mm^3 (n = 8 for each group). The mice xenografted with H22 hepatocarcinoma were treated with GPM/DOX and DOX with an equivalent DOX dose of 5.0 mg kg^{-1} per body weight, and normal saline as control by intravenous administration on Day 0, 4, and 8. The tumor volume was calculated by using Equation (3).

$$V\,(\text{mm}^3) = \frac{L \times S^2}{2} \qquad (4)$$

In Equation (3), L and S (mm) were the largest and smallest diameters of tumor, respectively.

1.2.10. Histopathological and immunohistochemical analyses

After all the treatments, the tumors were collected and cut into 5 μm slices. The slices were stained with hematoxylin and eosin (H&E) and immunohistochemistry (*i.e*., Caspase-3 and PCNA), respectively. The same pre-treatment was carried out as in Section 2.6, placed in a coverslip each well before the start of experiment. At the same time interval, the slices were roasted at 60 °C for 2 h, then deparaffinized twice with xylene for 20 min, and xylene was washed with 100% to 60% ethanol for 10 min each. The immunohistochemical stainings (*i.e.*, Caspase-3 and PCNA) were performed according to the routine method as follows: i) antigen retrieval; ii) serum closure; iii) adding primary antibody; iv) adding second antibody. The photos were taken by confocal laser scanning microscopy (CLSM; Carl Zeiss, LSM 780, Jena, Germany). The H&E microimages were taken by the microscope (Nikon Eclipse *Ti*, Optical Apparatus Co., Ardmore, PA). Three observation fields were repeated to get an average value of relative necrosis area with the area of total observation field as "100%". The immunohistochemical microimages were taken by CLSM. The optical density of control group was defined as "1", and the relative optical density was calculated by the optical density ratio of sample and control groups. The ImageJ software was used in the above operation for analyzing microimages.

1.2.11. Statistical Analysis

All tests were implemented at least three times, and the data were expressed as mean ± standard deviation (SD). Statistical analysis was performed using SPSS 21.0 for Windows (SPSS Inc., Chicago, IL, USA). $^{*}p < 0.05$ was considered statistically significant, and $^{**}p < 0.01$ and $^{\#}p < 0.001$ were considered highly significant.

2. Supplementary figures

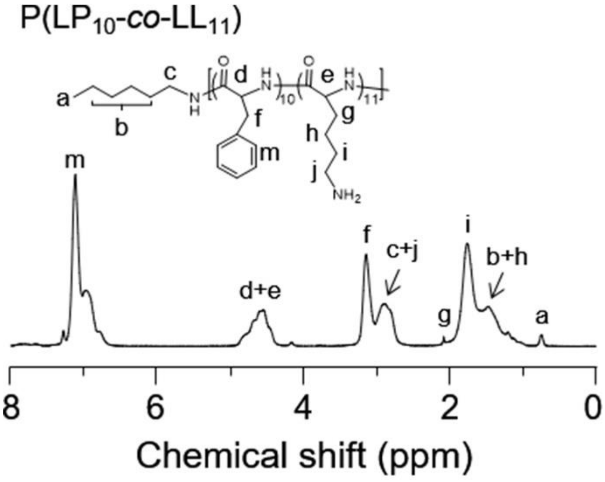

Figure S1 ^{1}H NMR spectra of P(LP$_{10}$-*co*-LL$_{11}$) (in TFA-*d*).

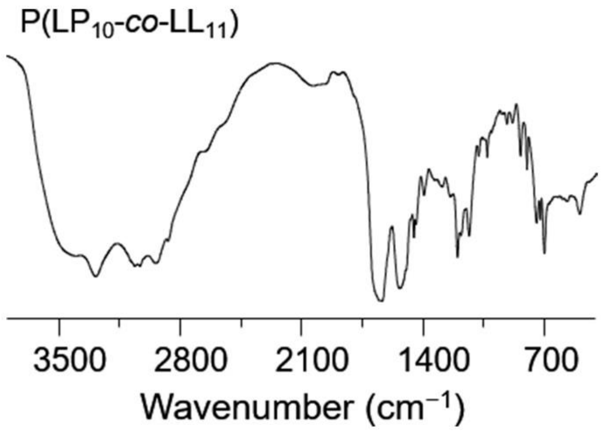

Figure S2 FT-IR spectra of P(LP_{10}-*co*-LL_{11}).

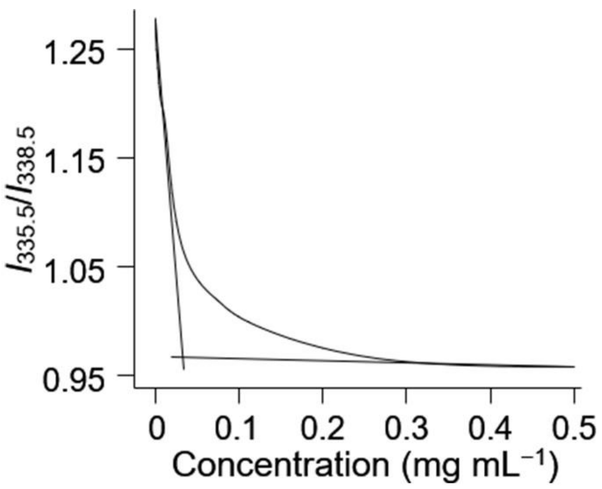

Figure S3 CMC of GPM.

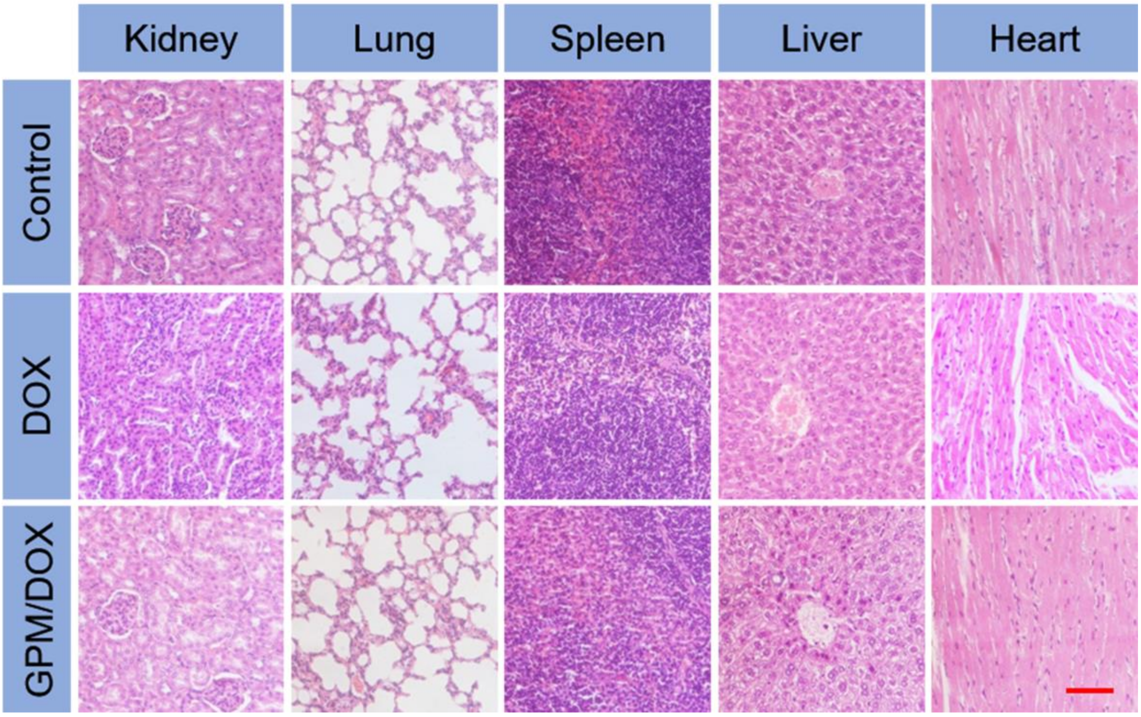

Figure S4 Histological analyses of main organ from H22-xenografted mice after treatment with free DOX, GPM/DOX, or normal saline (as control). Scale bar = 100 μm.
